# Neuronal message passing using Mean-field, Bethe, and Marginal approximations

**DOI:** 10.1038/s41598-018-38246-3

**Published:** 2019-02-13

**Authors:** Thomas Parr, Dimitrije Markovic, Stefan J. Kiebel, Karl J. Friston

**Affiliations:** 1 Wellcome Centre for Human Neuroimaging, Institute of Neurology, University College London, London, WC1N 3BG UK; 2 Chair of Neuroimaging, Psychology Department, Technische Universität Dresden, Dresden, Germany

## Abstract

Neuronal computations rely upon local interactions across synapses. For a neuronal network to perform inference, it must integrate information from locally computed messages that are propagated among elements of that network. We review the form of two popular (Bayesian) message passing schemes and consider their plausibility as descriptions of inference in biological networks. These are variational message passing and belief propagation – each of which is derived from a free energy functional that relies upon different approximations (mean-field and Bethe respectively). We begin with an overview of these schemes and illustrate the form of the messages required to perform inference using Hidden Markov Models as generative models. Throughout, we use factor graphs to show the form of the generative models and of the messages they entail. We consider how these messages might manifest neuronally and simulate the inferences they perform. While variational message passing offers a simple and neuronally plausible architecture, it falls short of the inferential performance of belief propagation. In contrast, belief propagation allows exact computation of marginal posteriors at the expense of the architectural simplicity of variational message passing. As a compromise between these two extremes, we offer a third approach – marginal message passing – that features a simple architecture, while approximating the performance of belief propagation. Finally, we link formal considerations to accounts of neurological and psychiatric syndromes in terms of aberrant message passing.

## Introduction

Recent work in theoretical neurobiology calls on the notion that the brain performs Bayesian inference^1–5^. This view treats perceptions as hypotheses about the causes of sensations^6,7^. Under this perspective, perceptual inference is the accumulation of evidence to confirm or refute various explanations for sensory data. As neuronal processing relies upon local signalling, the form of the inferences performed by the brain must involve the passing of local messages^8^. Here, we compare two forms of Bayesian message passing that have been used to explain cognitive phenomena. We consider their plausibility as accounts of neural processing, with a special focus on the anatomy of neural architectures that could implement these schemes. This calls for a set of criteria by which the plausibility of each scheme can be evaluated. Ultimately, this requires an evaluation of the evidence for each alternative process afforded by neurobiological data, considering prior constraints upon neural systems. Our focus here is upon the latter, and within this upon two important criteria. First, the computational architectures required for neuronal networks to perform inference should be as simple as possible. This is motivated by the spatial and metabolic constraints upon biological systems, and by Occam’s razor (i.e. in trying to explain brain function, we should adopt the simplest explanation that is consistent with observed data). The second feature, which must be balanced against the first, is that these networks should be able to make accurate inferences about the causes of incoming sensory data.

This paper builds upon recent work that compares Bayesian message passing schemes in a simulated planning and decision making task^9^. The approach here complements this, but has a different focus. In this paper, our focus is upon the form and dynamics of the neuronal networks that are needed to perform inference under alternative message passing schemes. The novel aspects of this work include the specification of belief propagation in terms of a continuous gradient descent (for comparison with the dynamics previously used for variational message passing schemes^10^) and a neuronal network architecture that performs this gradient descent. This affords the opportunity to compare the dynamics of belief-updating under existing schemes. We then unpack a novel scheme – marginal message passing, and argue that this offers a plausible compromise between the two criteria (simplicity and performance) considered above.

Local message passing schemes make use of simple update rules that can be applied to a probabilistic generative model^11,12^. The simplicity of these update rules can be illustrated using probabilistic graphical models – specifically, using normal factor graphs^13–15^. We will leverage the flexibility of these graphs in representing probabilistic models: this serves to illustrate that many common statistical inference procedures may be performed by passing local messages on factor graphs. However, different message passing algorithms differ in the approximate solution they provide – and in the computational complexity of the scheme. An interesting question then arises; which scheme might explain the ability of biological neural networks to perform statistical procedures such as blind source separation^16^. While we focus upon a hidden Markov model for illustrative purposes (as these have been used extensively in modelling perceptual inference e.g.^17–19^), the discussion here generalises to other generative models.

## Factor Graphs

We begin with an overview of inferential message passing, before specifying the message passing rules in detail. We consider plausible neuronal network architectures that could implement these schemes, and simulate the associated inferences. Finally, we consider the implications of each of these schemes for pathologies of neural computation.

**Figure 1:**
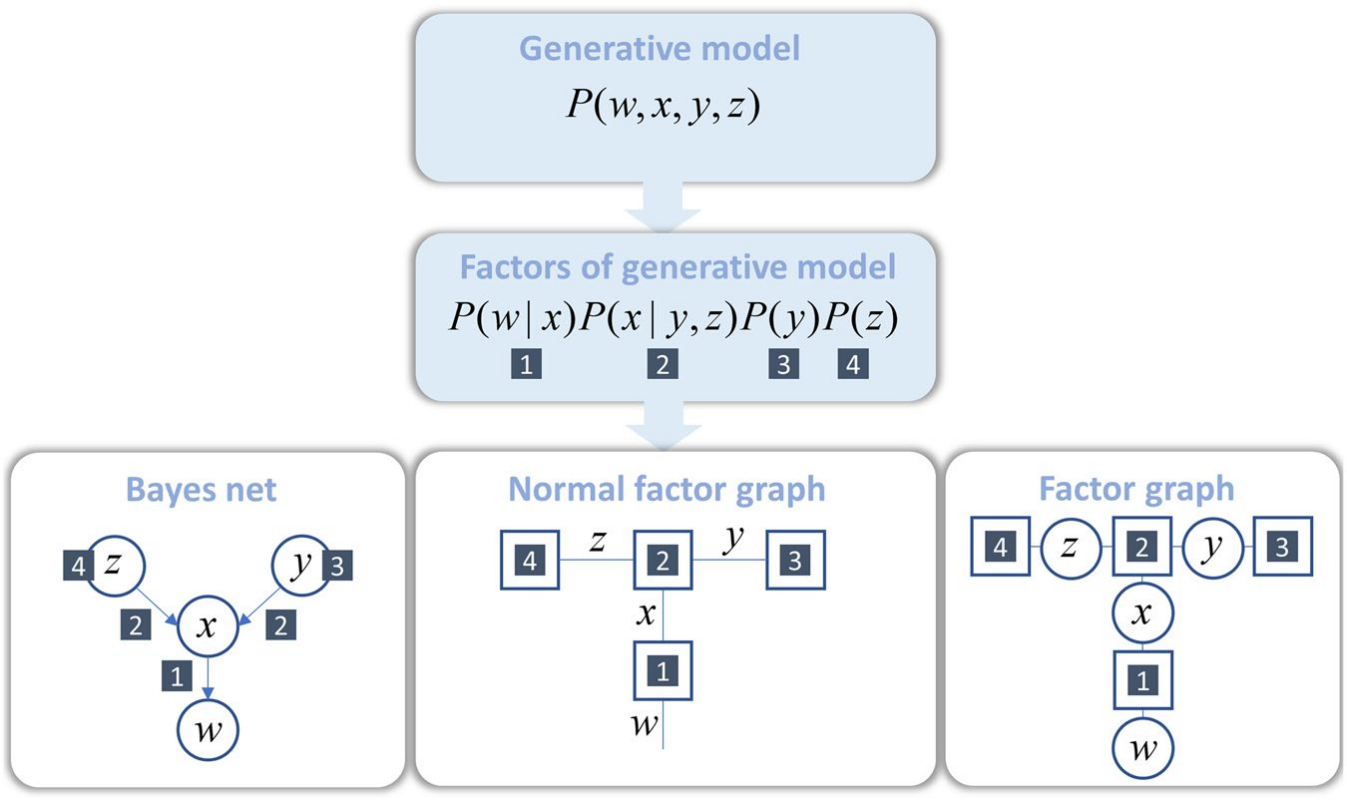
A graphical representation of a probabilistic model. For a generative model, expressed as a joint probability distribution, it is possible to write down the associated factor graph by following a few simple steps. First, the model may be expressed in terms of the factors (prior and conditional distributions) that make up the joint probability. Square nodes are then associated with each of these factors. These nodes are connected whenever they are functions of the same random variable. The result is known as a normal factor graph^13,20^. For comparison, we present the same generative model expressed according to two alternative graphical representations. The Bayes net shown on the left places random variables in circular nodes and connects these with arrows corresponding to the conditional distributions. An alternative factor graph representation is shown on the right. This combines the normal factor graph formalism with that of the Bayes net; incorporating both factor and variable nodes. For the rest of this paper, we adopt the normal factor graph formalism as this provides a natural way to think about the form of local message passing.

**Figure 2:**
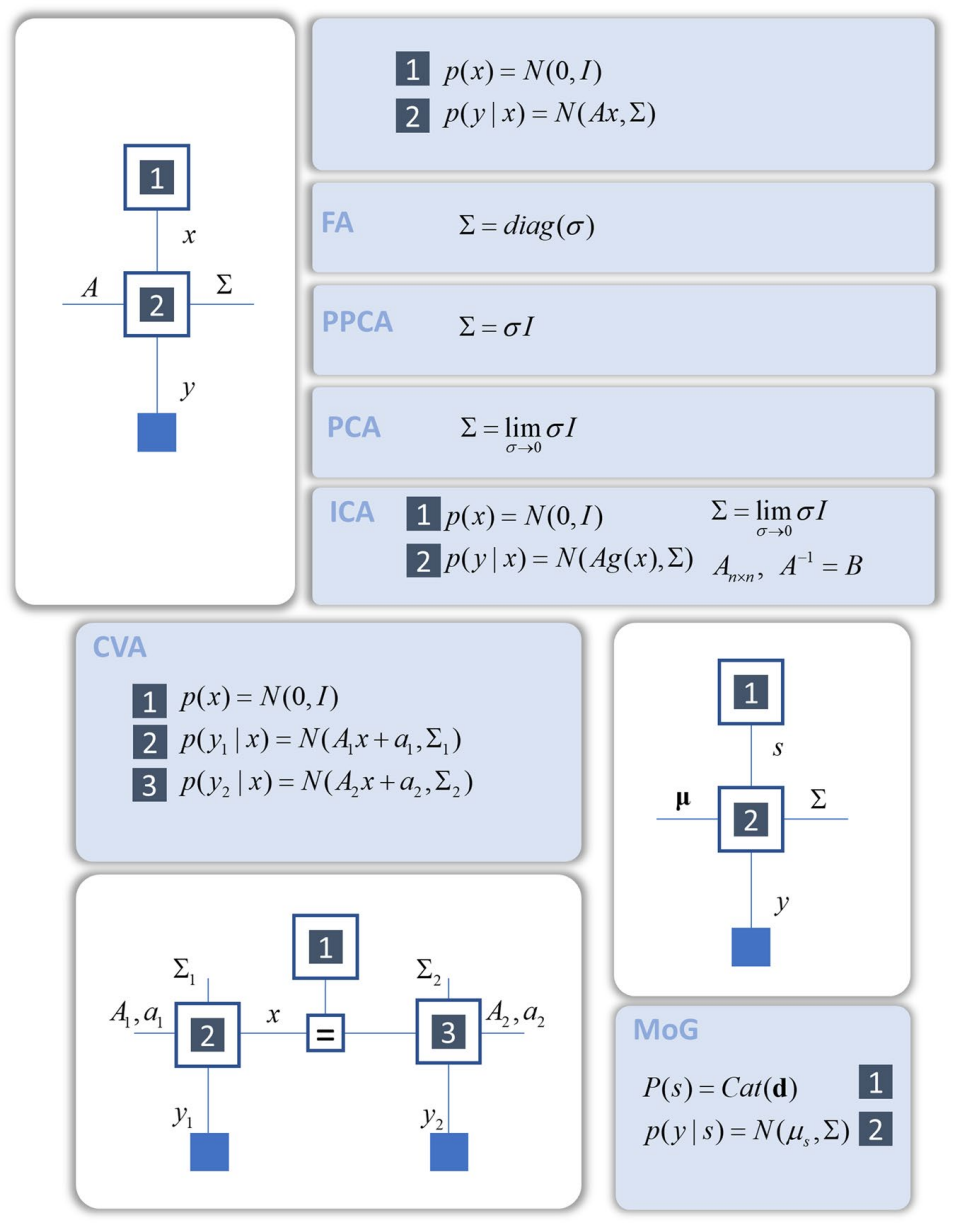
Commonly used (static) generative models as factor graphs. This figure illustrates the generative models that underwrite many common inferential procedures. In each factor graph, small blue squares indicate observable data, while squares with an equality sign relate their adjoining edges via a delta function factor (shown explicitly in lower right inset in Fig. 3 - Hidden Markov model). Numbered squares relate factors to those in the probabilistic models in the blue panels. This Figure illustrates static models of the sort that underlie factor analysis (FA), probabilistic principle component analysis (PPCA), and principal component analysis (PCA)^21^. Each of these dimensionality reduction techniques relies upon the same generative model, but with different assumptions about the covariance structure of latent causes or sources. Adding in non-linear functions allows this generative model to be extended to incorporate independent component analysis (ICA)^94^, while using two different linear transforms leads to probabilistic canonical variates analysis (CVA)^95^. Incorporating discrete random variables gives mixture models including mixtures of Gaussians (MoG), which form the basis of many clustering algorithms^96^. The notation *N* indicates a normal (Gaussian) distribution, while *Cat* means a categorical distribution.

**Figure 3:**
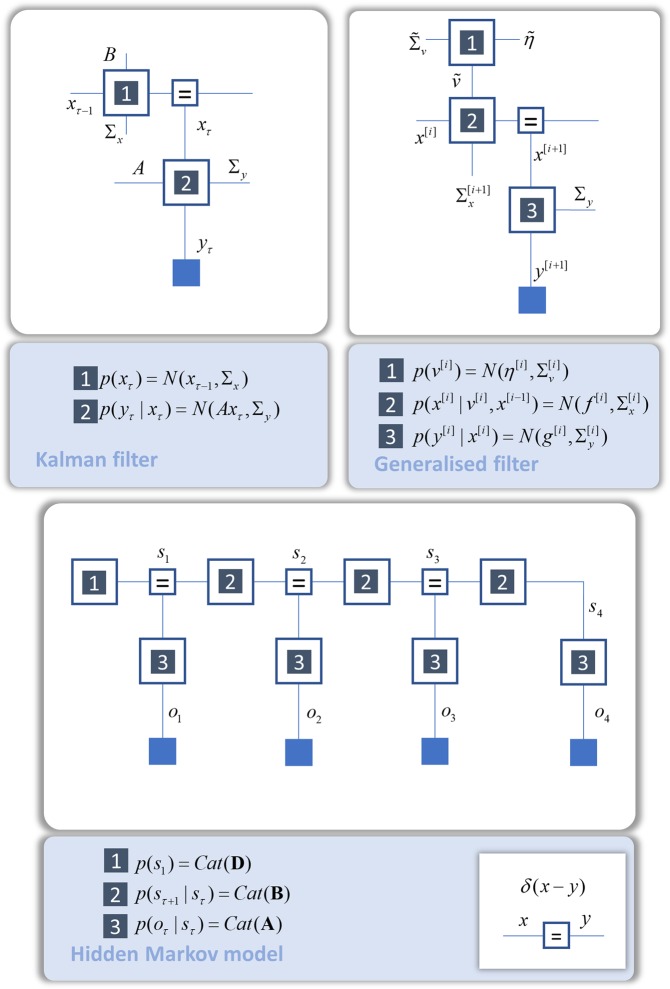
Commonly used (dynamic) generative models. This shows the dynamic generative models that support Kalman filtering^97^ and generalised filtering^98^ (the basis of predictive coding schemes^99^ and inversion of dynamic causal models^100^). A discrete state space model that exhibits temporal dynamics is a hidden Markov model. This is the generative model we will take as our example for the remainder of this paper and, for this reason, we have expressed this in full, with a sequence of transitions over time. The notation *N* indicates a normal (Gaussian) distribution, while *Cat* means a categorical distribution.

To map a probabilistic generative model to a factor graph, we follow the steps illustrated in Fig. 1 ^20^. We start with a generative model that expresses a joint probability distribution over all the random variables in that model. We then factorise the joint probability distribution to show the conditional dependencies implied by the model. Each factor is represented graphically by a square node. If two factors are functions of the same random variable, we connect these factors with an edge representing that variable. Any probabilistic generative model can be specified in this way^13^. To illustrate the flexibility of factor graphs Figs 2 and 3 provides factor graph formulations of some commonly used generative models in data analysis and machine learning^21^. Inference about any of these models can be performed through local message passing algorithms.

By message passing, we mean that each factor (square node) can synthesise the information coming in from one (or more) edge, and pass on this information in the form of a message along another edge. It is interesting to consider the relationship of this formal description of inference as message passing, and the informal notion that one part of the brain may communicate with another by sending a message. For the purposes of this paper, we equate the two, and associate the passing of messages between populations of neurons with inferential messages. These populations may be within a brain region, or may sit in different cortical areas. If the latter, the factor across which messages are passed is associated with the white matter tracts containing the axons that enable inter-areal communication.

The connection between message passing on a factor graph and effective connectivity in the brain may seem a little difficult to intuit, in relation to some theoretical accounts of cortical communication. Communication between brain areas is sometimes framed in terms of oscillatory processes, where coherence of oscillations determines the influence of one set of neurons over another^22^. While the link between this and a message passed across a given factor may not be immediately apparent, the two may be reconciled if we treat the coherence as parametrising the precision (inverse variance) associated with this factor. If we imagine the connection between two brain regions represents factor 1 in Fig. 1, greater coherence between those regions representing *x* and those representing *w* would enhance the precision of *P*(*w*|*x*), increasing the amount of information transmitted from factor 1 to the *x-*edge following an observation *w*.

Figure 2 shows the generative models that underwrite inferences about static latent variables. This includes dimensionality reduction techniques such as factor analysis and principal component analysis. From a generative modelling approach, these may be thought of as inferences about a low dimensional latent variable that generates relatively high dimensional data (with different assumptions about the covariance structure of this process). Similarly, independent components analysis, used to separate out data into different sets of causes, may be thought of as inferring the parameters of the mapping from a non-linear transform of a latent variable to data of the same dimension. Canonical variates analysis, a technique used to find linear transforms of two sets of multivariate data that render them maximally correlated, may be thought of as inferring the set of hidden states that best explain (i.e. could have generated) both datasets. Finally, clustering procedures, which are used to separate data into distinct clusters, are often based upon a ‘mixture-of-Gaussians’ generative model, that assumes data are generated from several different Gaussian distributions. Operations of this sort are important in inferring (and learning) the structure of our environment from sensory data. By framing these operations in terms of probabilistic inference, we can express them as local message passing procedures across appropriate factor graphs.

The procedures above may underwrite inferences the brain can draw about unchanging aspects of its environment. However, much of our environment is not static. As such, the brain must also make use of models like those of Fig. 3 that account for temporal dynamics. In the following, we focus upon Hidden Markov Models, as these provide a simple example of a dynamical generative model. Although we have chosen to illustrate the ideas in this paper using this example, we do not mean to imply that this is the best or only form of generative model used by the brain. Figures 2 and 3 make the point that, if the brain uses a particular local update rule defined on a factor graph, a whole range of inferential operations may be performed simply by applying these local rules to alternative factor graphs.

## Two Bayesian Message Passing Schemes

Bayesian message passing schemes work by passing messages from factor nodes (computed from the information at edges adjoining to that node) to each edge. The two messages arriving at each edge are multiplied together to obtain the posterior probability associated with the random variable at that edge. While these methods have found applications in engineering and machine learning^23,24^, we focus upon their biological implications. In the following, we consider two sorts of message. The first are those associated with belief propagation^25,26^. Often referred to as the ‘sum-product’ approach, belief propagation is a method used to perform exact Bayesian inference for marginal distributions on acyclic graphical models and approximate inference on cyclic graphs.

Belief propagation is at the heart of the circular inference account of neuronal computation^4,27^. Circular inference offers a biological implementation of belief propagation, and derives its name from the circular patterns of inhibitory connections it requires. This scheme additionally underwrites some theoretical accounts of the anatomy of cortical micro-circuitry^28^, and has been implemented in populations of simulated (spiking) neurons^15,29^.

A generic account of brain function that subsumes the Bayesian brain – and various forms of predictive processing such as predictive coding – is active inference^30^. This account derives from the imperative for living creatures to maximise Bayesian model evidence or, more simply, engage in self-evidencing^31^. This is equivalent to minimising their variational free energy^3^. One process theory associated with active inference^32^ proposes that communication between populations of neurons occurs through an architecture based upon variational message passing^12,33^.

Both belief propagation and variational message passing have had some success in reproducing aspects of cognitive function, e.g.^4,27,34–39^ but lead to rather different interpretations of false inference in neurological and psychiatric disorders.

## Inferential Message Passing

To illustrate the concepts we describe here, we use a Hidden Markov Model (HMM). This is a ubiquitous discrete state space model that also forms an important part of a Markov Decision Process. We chose the HMM as a showcase example for this study as it represents an important class of generative models used both in reinforcement learning^40,41^ and active inference^19,30,36^. Both techniques have been used when modelling behaviour and, in general, are suited to describe processes that evolve through time – something that is crucial for biological (as well as robotic^42^) systems. The inferential message passing in an HMM takes a simple form that is related to schemes used in engineering, such as the Baum-Welch (forward-backward) algorithm^23^. The key aspect of these generative models is that hidden states are represented at each point in time over a sequence of outcomes. In other words, the hidden state at the beginning of a sequence is distinct from the same state at the end. Although we have selected an HMM for illustrative purposes, the two message passing methods we review here are applicable to any probabilistic generative model.

Figure 3 (lower half) shows the form of an HMM as a normal factor graph^10,13,14,33^. This is a representation of a joint probability distribution in terms of its factors. It involves two types of random variable – observable outcomes (${o}_{\tau }$) and hidden states (${s}_{\tau }$). Hidden states evolve through time in a Markov chain. This means that each state depends only upon the state at the previous time. At each time, hidden states give rise to an outcome. The sparsity of conditional dependencies in this (and other) generative models allows for efficient local message passing schemes to be derived. This is because the messages used to compute beliefs about a variable come only from the constituents of the variable’s Markov blanket^11^. The Markov blanket of a given hidden state in an HMM contains the state in the immediate past, the state in the immediate future, and observable data at the present.

**Figure 4:**
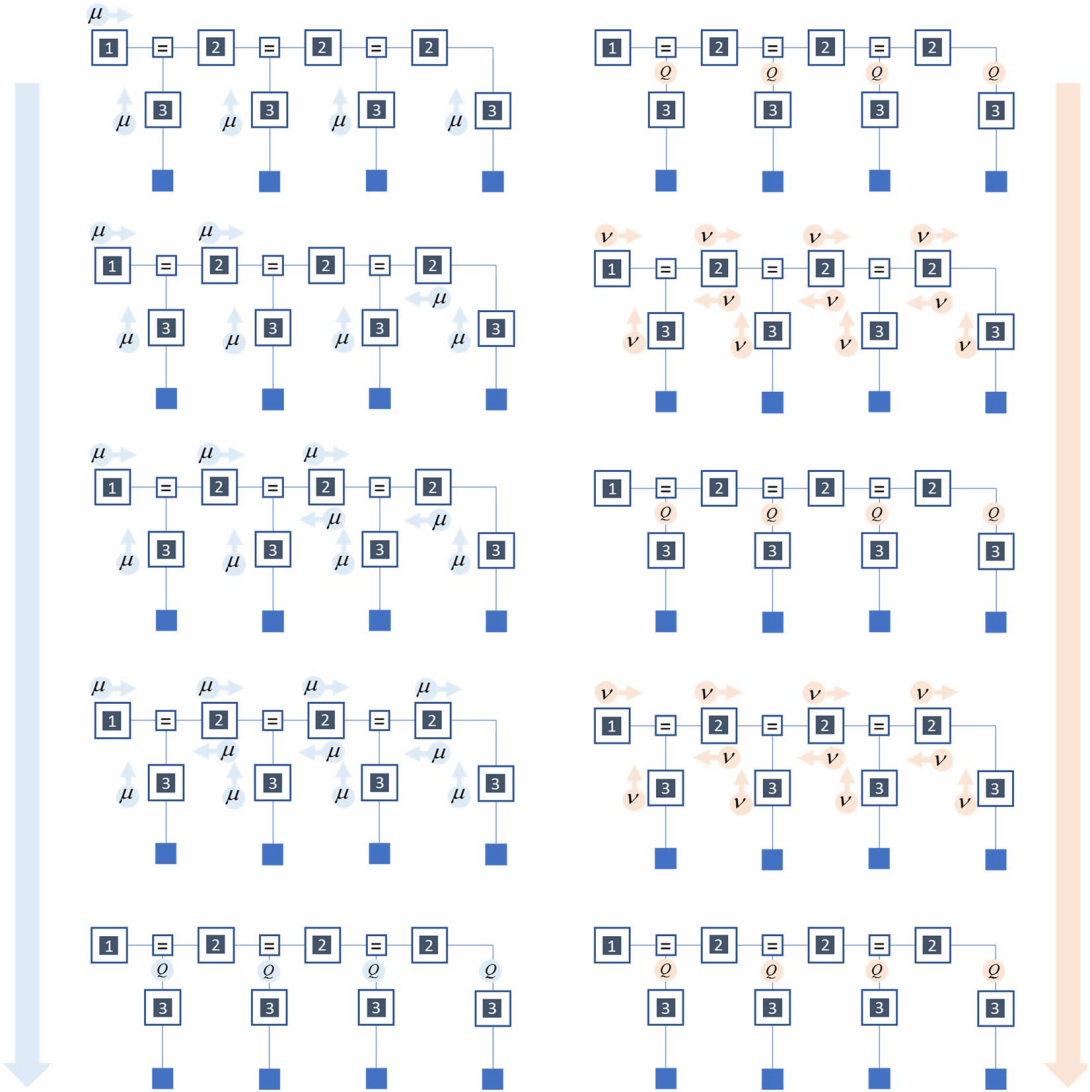
Message passing in a hidden Markov model. This schematic illustrates the scheduling of belief propagation (left) and variational message passing (right) in a hidden Markov model. Each row shows a single step in a round of message passing, ordered from top to bottom. Under belief propagation, for a message to be sent across a factor to an edge, the factor requires all of the other adjoining edges to provide a message. Initially, in the first step, this is only true for likelihood factors (that compute their message from the data), and priors (that are associated with just one edge). This enforces a strict scheduling that starts with the computation of messages at the extremities of the graph. More proximal factors then use these messages from the extremes to compute their own. Eventually, when all factors have passed their message, the incoming messages to each edge can be combined to compute the marginal posterior belief (*Q*) about the associated random variable (last row). For directed acyclic graphs, one round of message passing is sufficient. For cyclic graphs, multiple rounds may be required. In contrast, variational message passing computes messages from the current beliefs associated with each edge, and not from other incoming messages. This means that variational message passing simply alternates between message passing (of all messages in parallel) and updating posterior expectations.

This is illustrated in Fig. 4, where messages are indicated as arrows across factor nodes (large squares) to the edges (lines connecting factors) that represent the random variables. Each message can be computed from locally available information. The normalised product of incoming messages to an edge is the approximate posterior probability distribution over the random variable represented by that edge. The following sections overview two established message passing schemes – *belief propagation* and *variational message passing*. In addition, we introduce a third scheme – *marginal message passing* – that combines some of the key advantages of the previous two. We consider biologically plausible neuronal networks that could realise these schemes and simulate their behaviour when presented with sequential observations.

## Belief Propagation

Sum-product belief propagation arises naturally in directed acyclic graphs^25^ but can also be applied to cyclic graphs as the belief update rules correspond to the fixed points of the Bethe free energy^26^, see the appendix for details. The message passing can be expressed in the following way (with messages *μ* used to compute approximate posterior beliefs *Q* about states *s* at time *τ*)

1 $$\begin{array}{rcl}Q({s}_{\tau }) & \approx  & P({s}_{\tau }|\tilde{o})\propto P({o}_{\tau }|{s}_{\tau })\sum _{{s}_{1},{s}_{2},\ldots {s}_{\tau -1}}P({s}_{t < \tau },{o}_{t < \tau })\sum _{{s}_{\tau +1},{s}_{\tau +2},\ldots {s}_{T}}P({s}_{\tau },{s}_{t > \tau },{o}_{t > \tau })\\ Q({s}_{\tau }) & \propto  & {\mu }_{A}({s}_{\tau })\cdot {\overrightarrow{\mu }}_{B}({s}_{\tau })\cdot {\overleftarrow{\mu }}_{B}({s}_{\tau })\\ {\mu }_{A}({s}_{\tau }) & = & P({o}_{\tau }|{s}_{\tau })\\ {\overrightarrow{\mu }}_{B}({s}_{\tau }) & = & \sum _{{s}_{\tau -1}}P({s}_{\tau }|{s}_{\tau -1}){\overrightarrow{\mu }}_{B}({s}_{\tau -1}){\mu }_{A}({s}_{\tau -1})\\ {\overleftarrow{\mu }}_{B}({s}_{\tau }) & = & \sum _{{s}_{\tau +1}}P({s}_{\tau +1}|{s}_{\tau }){\overleftarrow{\mu }}_{B}({s}_{\tau +1}){\mu }_{A}({s}_{\tau +1})\end{array}$$

The first line of this equation uses an approximate equality, as *Q* is not always equal to the marginal posterior (although it is in the absence of cycles in a graph). The second line expresses a proportional relationship, as the product of messages needs normalisation. For consistency with the HMM shown in Fig. 3, we use a subscript A to indicate the messages being passed across a likelihood factor, and subscript B to indicate those passed across those factors representing transition probabilities (with a right-pointing arrow indicating a message derived from beliefs about the past, and a left-pointing arrow indicating a message from the future). One way to think about this scheme is that a message from a given region of the factor graph is the partition function of that region^43^. Each partition function is computed using the partition function of a sub-region within that region, and so on. The recursive computation of these messages would be problematic for a network of neurons representing marginal beliefs, as messages are derived from other messages to an edge (neuronal population), not from the marginal belief (neuronal activity) itself. This imposes strict constraints on the scheduling of message passing as illustrated in Fig. 4, where a factor does not pass a message on to a given edge until it has received messages from all the other edges connected to it. Technically, this constraint can be removed by using a ‘loopy’ belief propagation scheme^44^, or equivalently to rearrange the equations above so that messages depend upon the marginal^4^.

2 $${\overrightarrow{\mu }}_{B}({s}_{\tau })\propto \exp \,(\mathrm{ln}\,Q({s}_{\tau })-\,\mathrm{ln}\,{\mu }_{A}({s}_{\tau })-\,\mathrm{ln}\,{\overleftarrow{\mu }}_{B}({s}_{\tau }))$$

To retain the conditional dependencies of the generative model, we update the marginals through the following equation (which is obtained from the second line of Equation 1 by substituting in the message definitions on the final three lines)

3 $$Q({s}_{\tau })\propto \exp \,(\mathrm{ln}\,P({o}_{\tau }|{s}_{\tau })+\,\mathrm{ln}\,{E}_{{\overrightarrow{\mu }}_{B}({s}_{\tau -1}){\mu }_{A}({s}_{\tau -1})}[P({s}_{\tau }|{s}_{\tau -1})]+\,\mathrm{ln}\,{E}_{{\overleftarrow{\mu }}_{B}({s}_{\tau +1}){\mu }_{A}({s}_{\tau +1})}[P({s}_{\tau +1}|{s}_{\tau })])$$

**Figure 5:**
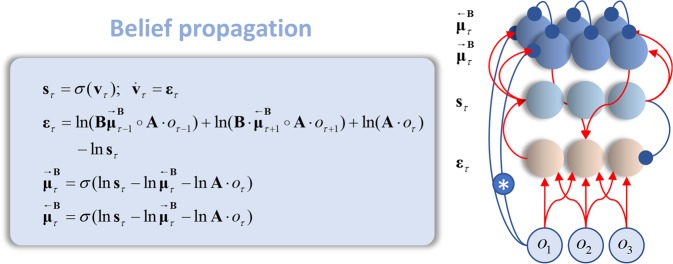
Belief propagation as neuronal message passing. The equations on the left show the form of belief propagation (Equations 2 and 3) when expressed in a neuronally plausible form. These equations are written in terms of the sufficient statistics of the probability distributions and auxiliary variables representing prediction errors (${{\boldsymbol{\varepsilon }}}_{\tau }$) and membrane potentials (${{\bf{v}}}_{\tau }$). The softmax ($\sigma$) functions act as neuronal transfer functions, converting presynaptic potentials to firing rates (${{\bf{s}}}_{\tau }$), which represent the sufficient statistics of the posterior beliefs. Forwards and backwards messages across the transition factors (${\bf{B}}$) are written as ${\overrightarrow{{{\boldsymbol{\mu }}}_{\tau }}}^{{\bf{B}}},{\overleftarrow{{{\boldsymbol{\mu }}}_{\tau }}}^{{\bf{B}}}$ respectively. Red indicates an excitatory connection, while blue is inhibitory. The starred connection represents the subtraction of the ascending message from that passed on to other neuronal populations. This plays the role of an ascending ‘loop’, as in circular inference accounts of neuronal computation^4,37^. The analogous descending loops are the inhibitory connections between the neurons representing messages in opposite directions. This formulation assumes that the neurons representing the messages have much shorter time constants than those representing marginal beliefs, allowing the former to be ‘enslaved’ by the latter^15,101^. Although omitted here (and in later figures) for simplicity, the normalisation induced by the softmax functions could be mediated via recurrent inhibitory connections within a layer of neurons.

The ‘expectation’ notation is used heuristically here, as the messages are not probability distributions. We use this notation to mean a summation, where every element of the sum is weighted by the subscripted term (i.e. a linear combination of the terms within the expectation). Note that we could have written the above more simply as a product of the terms within the logarithms (similar to the second line of Equation 1). However, we have opted to express this as an exponential of the sum of three logarithms for consistency with the form of the equations for the other message passing schemes presented later. Once we have expressed the equations in this form, the marginals begin to play an important part in the message passing. Expressing the updates in the form of a gradient descent, we arrive at neuronally plausible updates as shown in Fig. 5. To obtain these equations we compute an error term (**ε**) that is the difference between (the log of) the current estimate of the posterior probability (**s**) and the right hand side of Equation 3. We then construct a differential equation that changes **s** and has an **ε** of zero at its fixed (attracting) point. The softmax (normalised exponential) functions have a high degree of biophysical plausibility, as the density dynamics of spiking neuron populations have a similar form^45^, where synaptic input is converted into firing rates that can be propagated along axons to other neural populations. For an interpretation of belief propagation in terms of spiking neurons, see^46,47^. The probability matrices now become connectivity matrices, lending a clear biological interpretation to the inferential equations above. Note that the gradient descent described by the differential equation occurs over a faster time scale than the frequency at which observations change. Biologically, this is consistent with things like the fast neuronal processing (gradient descent) that intervenes between saccadic eye movements (mediating changes in sensory input).

The use of separate populations of neurons that represent messages and marginals resembles previous accounts of belief propagation in neuronal networks^15,48^. An alternative is that one abandons any explicit representation of marginal probabilities, and that neurons represent the messages^15,49^ only. This interpretation has two drawbacks. First, it runs into the same scheduling issues described above. Second, explicit representations of the marginal beliefs are essential in performing inferential operations including model comparison, model selection, and model averaging. This is because these operations require evaluation of approximations to model evidence (and expected model evidence) that depend upon these posteriors. Importantly, to properly estimate model evidence as a minimum of the Bethe free energy (see below) besides the singleton marginals, a neuronal network implementing belief propagation would also have to represent the pairwise marginals, which would add additional degrees of complexity to the network illustrated in Fig. 5. Model comparison, model selection, and model averaging are thought to underwrite the evaluation of behavioural policies that support active engagement with the sensorium^50,51^ and inference with hierarchical models^10^.

## Variational Message Passing

Variational message passing takes a superficially similar form to belief propagation. Marginals of the posterior distribution are computed by the product of messages from neighbouring factors^12,33^.

4 $$\begin{array}{lll}Q({s}_{\tau }) & \propto  & {\nu }_{A}({s}_{\tau })\cdot {\overrightarrow{\nu }}_{B}({s}_{\tau })\cdot {\overleftarrow{\nu }}_{B}({s}_{\tau })\\ \mathrm{ln}\,{\nu }_{A}({s}_{\tau }) & = & \mathrm{ln}\,P({o}_{\tau }|{s}_{\tau })\\ \mathrm{ln}\,{\overrightarrow{\nu }}_{B}({s}_{\tau }) & = & {E}_{Q({s}_{\tau -1})}[\,\mathrm{ln}\,P({s}_{\tau }|{s}_{\tau -1})]\\ \mathrm{ln}\,{\overleftarrow{\nu }}_{B}({s}_{\tau }) & = & {E}_{Q({s}_{\tau +1})}[\,\mathrm{ln}\,P({s}_{\tau +1}|{s}_{\tau })]\end{array}$$

**Figure 6:**
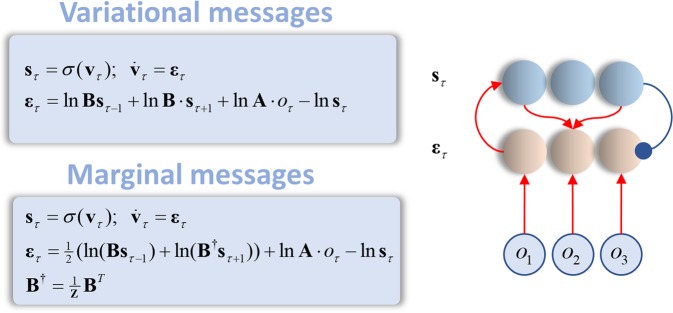
Variational (and marginal) message passing. The equations on the left show variational message passing (upper panel) and marginal message passing (lower panel) expressed as gradient descents on the variational (or marginal) free energy. These equations are implemented by the neuronal network shown on the right. Notably, this is much simpler than the network of Fig. 5, requiring fewer neurons and a simpler connectivity structure. The primary reason for the simplicity of this structure is that these schemes take into account the current marginal beliefs of adjacent variables. The messages do not need to be recursively computed from other messages, Fig. 5. This limits the number of auxiliary variables required. We have introduced the notation ${{\bf{B}}}^{\dagger }$ for the transpose of the transition matrix with normalised columns.

In contrast to belief propagation, the messages (*ν*) here are derived from the posterior marginal beliefs at each edge. This means that we do not need to wait for all of the incoming messages. Instead, we can iterate between computing messages (at all factor nodes in parallel) and updating posterior beliefs. Using a gradient ascent ensures we can combine these steps into a single differential equation, as in Fig. 6, without any need to manipulate the form of the messages as with belief propagation. This leads naturally to the simple neuronal network illustrated here; for which connectivity matrices are log probabilities. The structure in Fig. 6 forms part of the cortical microcircuit proposed for active inference in Markov decision processes^52^, and can be extended for generative models associated with precision parameters^53^, and for continuous state space models^10^.

If we used a generative model that contained *n* types of hidden state, that could take on *m* possible values, for *t* time-steps, the architecture of Fig. 6 would require 2 × *n* × *m* × *t* neuronal populations. On comparing this to the 4 × *n* × *m* × *t* populations required for the architecture of Fig. 5, it is clear that there is a substantial saving to adopting the architecture of Fig. 6 for any sizable generative model. Appealing to minimum wire length principles^54^ and taking note of the metabolic^55^ and therefore informational^56^ costs of individual neurons, this network has a substantial structural advantage over that given by belief propagation. However, dendritic computations^57^ may have the potential to rescue belief propagation in this regard; i.e., it is possible that forwards and backwards messages could be represented in different parts of the dendritic tree, eliminating the need for additional ‘message’ neurons.

## Marginal Message Passing

A third approach to inference combines the simplicity offered by variational message passing with the sophistication of belief propagation. This aims to approximate the messages from the former, but to do so using only the locally available marginal beliefs. This was first described as an alternative to variational message passing for active inference in Appendix C of^32^. Marginal message passing recapitulates the pattern from Equations 1 and 4, with marginal posteriors expressed as the product of messages (*η*) from their Markov blanket:

5 $$\begin{array}{lll}Q({s}_{\tau }) & \propto  & {\eta }_{A}({s}_{\tau })\cdot {\overrightarrow{\eta }}_{B}({s}_{\tau })\cdot {\overleftarrow{\eta }}_{B}({s}_{\tau })\\ \mathrm{ln}\,{\eta }_{A}({s}_{\tau }) & = & \mathrm{ln}\,P({o}_{\tau }|{s}_{\tau })\\ \mathrm{ln}\,{\overrightarrow{\eta }}_{B}({s}_{\tau }) & = & \tfrac{1}{2}\,\mathrm{ln}\,{E}_{Q({s}_{\tau -1})}[P({s}_{\tau }|{s}_{\tau -1})]\\ \mathrm{ln}\,{\overleftarrow{\eta }}_{B}({s}_{\tau }) & = & \tfrac{1}{2}\,\mathrm{ln}\,{E}_{Q({s}_{\tau +1})}[P({s}_{\tau }|{s}_{\tau +1})]\end{array}$$

Like belief propagation, the expectation sits within the logarithm. Like variational message passing, the messages are derived from adjacent marginals. A comparison with equation 4 shows that this approach implicitly assumes the following relation.

6 $$\tfrac{1}{2}\,\mathrm{ln}\,{E}_{Q({s}_{\tau -1})}[P({s}_{\tau }|{s}_{\tau -1})]\approx \,\mathrm{ln}\,\mathop{\underbrace{{E}_{P({s}_{\tau -1}|{o}_{1},{o}_{2},\mathrm{...}{o}_{\tau -1})}[P({s}_{\tau }|{s}_{\tau -1})]}}\limits_{{\overrightarrow{\mu }}_{B}({s}_{\tau })}$$

Intuitively, as $Q({s}_{\tau -1})$ approximates the posterior following all available observations, it will be more precise (i.e. have a lower Shannon entropy) than $P({s}_{\tau -1}|{o}_{1},{o}_{2},\mathrm{...}{o}_{\tau -1})$. This is because the latter represents a partial posterior that considers only past observations. Halving the log message computed using the approximate posterior reduces the precision of the resulting message, better approximating the belief propagation message. The motivation for using ½ will become more apparent when we describe the underlying free energy functional in the next section. However, it would also be possible to treat this corrective factor as a parameter that itself could be optimised in relation to data. Variational message passing fails to attenuate the precision of this message and leads to overconfidence in estimating posteriors, as we will illustrate below using simulations. The marginal approach retains the simplicity of the variational architecture but eludes this overconfidence issue. An interesting feature of this scheme is that backwards messages use transitions from the future to the past – something not seen in the other two approaches. We will unpack this in more detail in the following section.

## Model Evidence and Free Energies

Both variational message passing, and belief propagation can be shown to represent fixed points for approximations to model evidence^26^. In each case, these approximations take the form of free energy functions. In this section, we briefly outline the relationship between free energy and model evidence. We then specify the free energies that act as the landscapes upon which these inferential optimisations take place. In brief, the variational free energy (under the mean-field approximation) approximates model evidence using a relatively simple form for the approximate posterior, in which one assumes no interactions between random variables. For belief propagation, the Bethe approximation uses a more sophisticated approximate posterior which takes into account pairwise interaction, but under specific conditions sometimes found in cyclic graphs may lead to erroneous estimates of the free energy.

Given that belief propagation may be motivated as in Equation 1, it might seem a little redundant to additionally motivate it in terms of the Bethe approximation. However, the Bethe approximation is crucial in understanding how these different message passing schemes relate to one another – as all are free energy minimising schemes that maximise a lower bound on model evidence. It is also important in understanding the approximations that belief propagation makes in a general setting, and in justifying the generalisation of belief propagation to settings beyond acyclic graphs.

In a typical inference problem, one is interested in determining posterior beliefs over hidden states $\tilde{s}=({s}_{1},\ldots ,{s}_{T})$ given some set of observations $\tilde{o}=({o}_{1},\ldots ,{o}_{T})$ using Bayes’ rule

7 $$P(\tilde{s}|\tilde{o})=\frac{P(\tilde{o},\tilde{s})}{P(\tilde{o})}$$

For a general inference problem, the above relation is analytically intractable. First, the denominator on the right-hand side (also known as model evidence or marginal likelihood) can be only estimated using approximate numerical methods. Second, the posterior probability distribution $P(\tilde{s}|\tilde{o})$ might not have a known analytic form. Variational inference resolves these difficulties using the following approximate scheme for probabilistic inference: (i) Map the true posterior to a tractable parametric family of probability distributions $Q(\tilde{s})$. (ii) Find the approximate estimate of the true posterior at the minimum of a free energy approximation to the negative model evidence.

Model evidence is related to free energy through Jensen’s inequality^58^.

8 $$\mathop{\underbrace{-F}}\limits_{{\rm{Negative}}\,{\rm{Free}}\,{\rm{Energy}}}=\mathop{\underbrace{{{\rm{E}}}_{Q}\,[\mathrm{ln}\,\frac{P(\tilde{o},\tilde{s})}{Q(\tilde{s})}]\le \,\mathrm{ln}\,{{\rm{E}}}_{Q}\,[\frac{P(\tilde{o},\tilde{s})}{Q(\tilde{s})}]}}\limits_{\mathrm{Jensen}\text{'}s\,{\rm{inequality}}}=\mathop{\underbrace{\mathrm{ln}\,P(\tilde{o})}}\limits_{\mathrm{log}\,{\rm{evidence}}}$$

The first of these equations indicates that the (negative) free energy is a lower bound on the evidence for a generative model (known in machine learning as an evidence lower bound or ELBO).

We are concerned with the optimisation of this bound, that is, with finding $Q(\tilde{s})$ which minimises free energy. The minimum of the free energy corresponds to the best approximation to the true posterior and closest estimate of the model evidence, within the given family of probability distributions. A rearrangement of the terms in the left-hand side of the inequality above gives

9 $$\begin{array}{rcl}F & = & -\mathop{\underbrace{\mathrm{ln}\,P(\tilde{o})}}\limits_{{\rm{Evidence}}}+\mathop{\underbrace{{D}_{KL}[Q(\tilde{s})||P(\tilde{s}|\tilde{o})]}}\limits_{{\rm{Divergence}}}\\  & = & \mathop{\underbrace{-{E}_{Q}[\,\mathrm{ln}\,P(\tilde{o},\tilde{s})]}}\limits_{{\rm{Energy}}}-\mathop{\underbrace{H[Q(\tilde{s})]}}\limits_{{\rm{Entropy}}}\end{array}$$

The first line shows that the bound between the free energy and model evidence is the KL-Divergence between *Q* and the posterior distribution (i.e. the best approximation to the posterior is at the free energy minimum). The second shows the free energy expressed as an energy minus entropy (Shannon entropy of the approximate posterior). The minimum of the free energy *F* is obtained for $Q(\tilde{s})=P(\tilde{s}|\tilde{o})$, in which case the free energy is equal to the negative log-model evidence. However, a difficulty here is to find a good approximation to the true posterior which makes computation of both the energy and the entropy analytically tractable^59,60^.

The mean-field and the Bethe approximations choose different forms for the distribution *Q*. The mean-field approximation^61^ assumes fully factorised posterior probability (although the same principles apply to structured mean-field factorisations^33^).

10 $$Q(\tilde{s})=\prod _{\tau }Q({s}_{\tau })$$

The Bethe approximation is more nuanced, and accounts for the pairwise interactions between variables.

11 $$Q(\tilde{s})=\prod _{\tau }Q({s}_{\tau })\prod _{\tau ,\tau -1}\frac{Q({s}_{\tau },{s}_{\tau -1})}{Q({s}_{\tau })Q({s}_{\tau -1})}$$

Returning to the energy-entropy expression, we can write the variational free energy (for a HMM) as

12 $$\begin{array}{c}F=\mathop{\underbrace{-\sum _{\tau }({E}_{Q({s}_{\tau })Q({s}_{\tau -1})}[\,\mathrm{ln}\,P({s}_{\tau }|{s}_{\tau -1})]+{E}_{Q({s}_{\tau })}[\,\mathrm{ln}\,P({o}_{\tau }|{s}_{\tau })])}}\limits_{{\rm{Energy}}}\\ \,\,\,\,\,-\mathop{\underbrace{\sum _{\tau }H[Q({s}_{\tau })]}}\limits_{{\rm{Entropy}}}\end{array}$$

We then write the Bethe free energy (free energy under the Bethe approximation) as

13 $$\begin{array}{c}F=\mathop{\underbrace{-\sum _{\tau }({E}_{Q({s}_{\tau },{s}_{\tau -1})}[\,\mathrm{ln}\,P({s}_{\tau }|{s}_{\tau -1})]+{E}_{Q({s}_{\tau })}[\,\mathrm{ln}\,P({o}_{\tau }|{s}_{\tau })])}}\limits_{{\rm{Energy}}}\\ \,\,\,\,\,-\mathop{\underbrace{\sum _{\tau }(H[Q({s}_{\tau })]-{D}_{KL}[Q({s}_{\tau },{s}_{\tau -1})||Q({s}_{\tau })Q({s}_{\tau -1})])}}\limits_{{\rm{Bethe}}\,{\rm{entropy}}}\end{array}$$

Although the energy component of the Bethe free energy preserves pairwise interactions between temporally proximate states in the expectation, this makes the entropy term a little more complicated. We can express the Bethe entropy in terms of the entropy of the marginal factors, but then subtract the mutual information between these and the joint factors. This approximates the true entropy (entropy of the exact posterior) by taking into account only the pairwise interactions between the variables and ignoring any higher order dependencies. For directed acyclic graphs (of the sort considered in this paper), the Bethe energy is always exact and the Bethe entropy, although approximate, will always correspond to the entropy of the joint probability distribution $Q(\tilde{s})$.

However, for a cyclic graph the Bethe energy is approximate, and the Bethe entropy might return suboptimal estimates of the entropy of the joint posterior, under certain conditions. This can happen when the solutions that satisfy the relation between the singleton $Q({s}_{t})$ and pairwise marginals $Q({s}_{t},\,{s}_{t-1})$ are implausible for a given cyclic graph. Importantly, in such cases, the Bethe free energy might produce strange behaviour (e.g. convergence to a limit cycle or improbable configurations of the marginal posterior) and anomalous free energy estimates. To mitigate these issues, higher order approximations have been proposed that are based on cluster variational methods and the Kikuchi approximation^59,62,63^.

The reason for the differences in convergence behaviours in mean-field and Bethe approaches is related to the convexity (or non-convexity) of their respective free energy functionals, specifically the entropy terms. A negative entropy is a convex functional, with a positive curvature. However, the negative Bethe entropy has contributions from the negative pairwise (convex) and positive singleton (non-convex) entropies. While overlapping pairwise marginals are sufficient to characterise the posterior, the Bethe entropy will always be convex. If interactions between three or more variables contain information that cannot be captured in overlapping pairwise interactions, the singleton entropies could dominate the pairwise entropies in some parts of the free energy landscape, inducing non-convexities that impede convergence. The mean-field entropy is not subject to this problem, as it comprises only positive entropy terms. This is a slightly simplistic explanation, to aid intuition. Interested readers are referred to^60,64^ for more formal treatments of this issue.

Despite these convergence issues, the Bethe free energy is often a better approximation to the log evidence than the variational free energy (when a mean-field approximation is employed). Under the mean-field approximation both the energy and entropy terms are approximations of the energy and entropy terms that would be obtained by setting the approximate posterior equal to the true posterior. For this reason, we ideally want to make inferences that are as close as possible to those obtained using belief propagation. The marginal free energy offers a way to do this, while retaining the architecture of variational message passing. Marginal message passing (Equation 5) is the scheme obtained at the fixed point of the maringal free energy.

**Figure 7:**
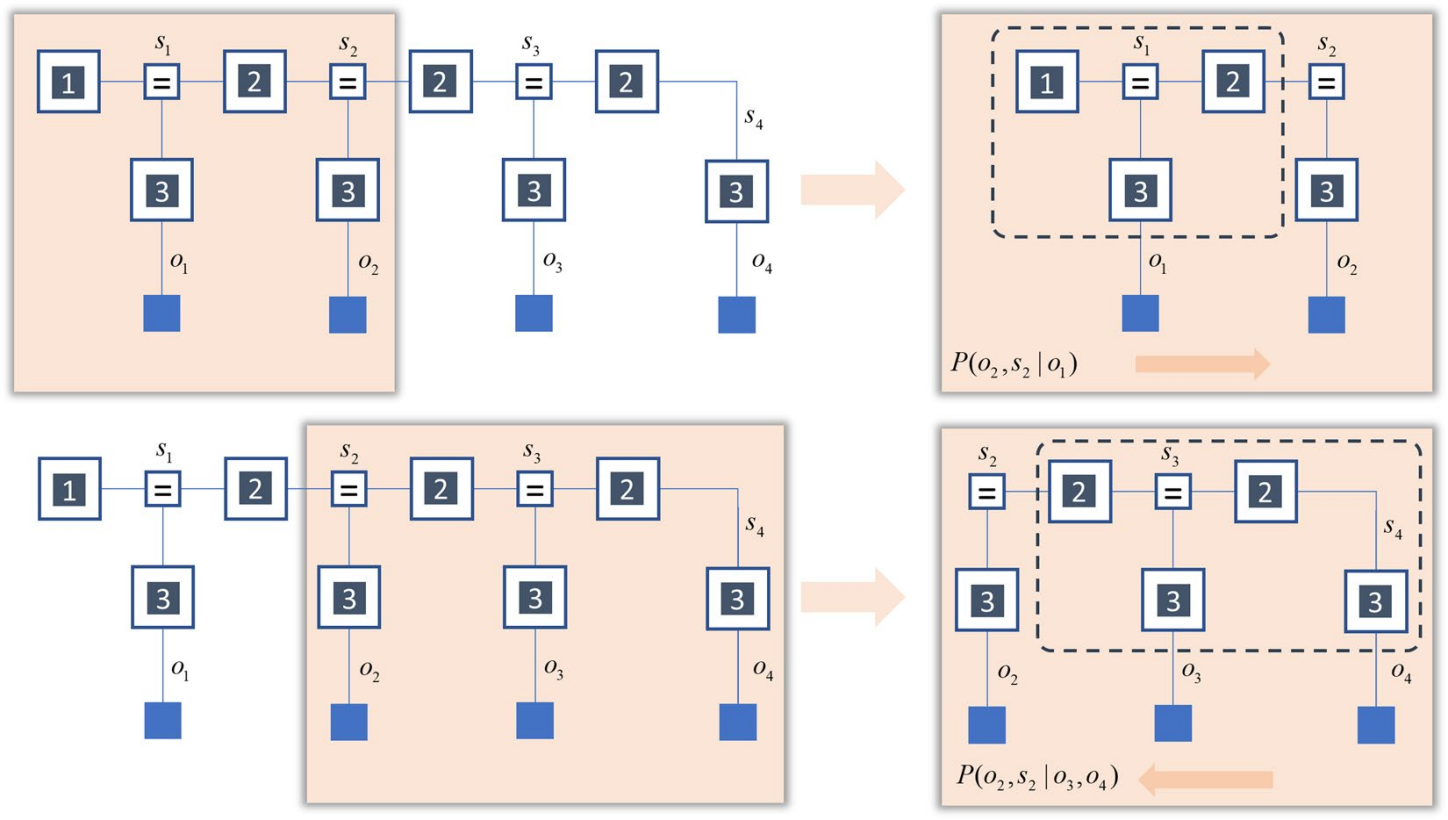
Marginal (forward-backward) models. This schematic illustrates the steps that motivate the marginal free energy. On the left, we show an HMM that is divided in two different ways. For the future part (lower row), we reverse the direction of the transition probabilities by normalising with respect to the earlier times. On the right, we take these partitioned generative models and sum over all variables within the dashed boxes. This leads to two, marginal, generative models – one that progresses from the past to the future, and one that reverses this. By approximating the free energies for each model, and mixing them in equal parts, we define a marginal free energy with empirical priors that are independently constrained by the future and the past.

Unlike the mean-field or Bethe approaches, marginal message passing makes no claim as to the form of the full posterior belief. Instead, it relies upon locally defined free energy functionals to optimise marginals of the posterior at each time, while remaining agnostic about how these combine to form a global free energy. Figure 7 illustrates the idea behind this functional. First, we divide the generative model into two overlapping parts – past and future – around the variable we wish to estimate. We then sum (or integrate) over all other hidden states. This leads to two marginal generative models, the first with an empirical prior derived from the past and the second with an empirical prior derived from the future.

14 $$\begin{array}{rcl}P({o}_{\tau },{s}_{\tau }|{o}_{1},\ldots {o}_{\tau -1}) & = & P({o}_{\tau }|{s}_{\tau })\mathop{\underbrace{{E}_{P({s}_{\tau -1}|{o}_{1},\ldots {o}_{\tau -1})}[P({s}_{\tau }|{s}_{\tau -1})]}}\limits_{P({s}_{\tau }|{o}_{1},\mathrm{...}{o}_{\tau -1})}\\ P({o}_{\tau },{s}_{\tau }|{o}_{\tau +1},\ldots {o}_{T}) & = & P({o}_{\tau }|{s}_{\tau })\mathop{\underbrace{{E}_{P({s}_{\tau +1}|{o}_{\tau +1},\ldots {o}_{T})}[P({s}_{\tau }|{s}_{\tau +1})]}}\limits_{P({s}_{\tau +1}|{o}_{\tau +1},\ldots {o}_{T})}\end{array}$$

To ensure that the empirical prior from the future sums to one, we have normalised the transition probabilities so that the future causes the past. This implies the HMM could be run in reverse, consistent with the conservation of probability mass. Although the empirical priors cannot be computed without resorting to the recursive approach of belief propagation, we can approximate these, to give the following (forwards and backwards) free energies.

15 $$\begin{array}{rcl}{F}_{F}(\tau ) & = & \mathop{\underbrace{-{E}_{Q({s}_{\tau })}[\,\mathrm{ln}\,P({o}_{\tau }|{s}_{\tau })+\,\mathrm{ln}\,{E}_{Q({s}_{\tau -1})}[P({s}_{\tau }|{s}_{\tau -1})]]}}\limits_{{\rm{Energy}}}-\mathop{\underbrace{H[Q({s}_{\tau })]}}\limits_{{\rm{Entropy}}}\\ {F}_{B}(\tau ) & = & \mathop{\underbrace{-{E}_{Q({s}_{\tau })}[\,\mathrm{ln}\,P({o}_{\tau }|{s}_{\tau })+\,\mathrm{ln}\,{E}_{Q({s}_{\tau +1})}[P({s}_{\tau }|{s}_{\tau +1})]]}}\limits_{{\rm{Energy}}}-\mathop{\underbrace{H[Q({s}_{\tau })]}}\limits_{{\rm{Entropy}}}\end{array}$$

We conjecture, but offer no proof for, the inequality:

16 $${F}_{B}(\tau )+{F}_{F}(\tau )\ge -{E}_{Q({s}_{\tau })}[\,\mathrm{ln}\,P({o}_{\tau },{s}_{\tau }|{\tilde{o}}_{\backslash \tau })]-H[Q({s}_{\tau })]$$

This suggests we can define an (approximate) marginal free energy as the mixture of forwards and backwards free energies.

17 $$F(\tau )=-\,{E}_{Q({s}_{\tau })}[\tfrac{1}{2}\,\mathrm{ln}\,{E}_{Q({s}_{\tau -1})}[P({s}_{\tau }|{s}_{\tau -1})]+\tfrac{1}{2}\,\mathrm{ln}\,{E}_{Q({s}_{\tau +1})}[P({s}_{\tau }|{s}_{\tau +1})]+\,\mathrm{ln}\,P({o}_{\tau }|{s}_{\tau })]-H[Q({s}_{\tau })]$$

This can then be optimised at each time-step. Minimising the marginal free energy can be thought of as applying variational filters in forwards and backwards directions and combining the results. This is subtly different to the mean-field and Bethe approaches, that each apply a single Bayesian smoother. The mixture in the marginal approach ensures we do not overestimate the precision of forwards or backwards messages, as could happen when optimising a mean-field posterior.

We hope that we will be able to provide a formal proof of Equation 16 in future work, and to be able to specify the conditions under which (if any) the inequality may fail. However, even in the absence of this, it is possible to motivate Equation 17 on heuristic grounds. The overconfidence of variational message passing depends upon the way in which beliefs about factors of the approximate posterior constrain one another. In Equation 17, the contribution of terms that depend upon other factors has been attenuated relative to those that do not mediate this influence. Notably, this means that the entropy of posterior beliefs (final term) offers a greater contribution to the free energy gradients than it would under a mean-field approach. This favours solutions with more uncertainty (i.e. minima with lower curvature in the free energy landscape^65^) than can be achieved through variational message passing; thereby, finessing the overconfidence problem.

Belief propagation represents the message passing scheme obtained at the stationary point of the Bethe free energy, variational message passing is the scheme found at the stationary point of the variational free energy, and marginal message passing represents the minimum of the marginal free energies at each time (see Appendix). On acyclic graphs they all act as lower bounds for the evidence for a model and could be utilised by a self-evidencing biological system. As such, all three forms of neuronal message passing are consistent with the principles that underwrite active inference^9^. The efficiency of these algorithms makes them especially suitable in biological setting, as they can ensure the minimisation of the free energy, and its time integral in an efficient manner.

## Simulations

**Figure 8:**
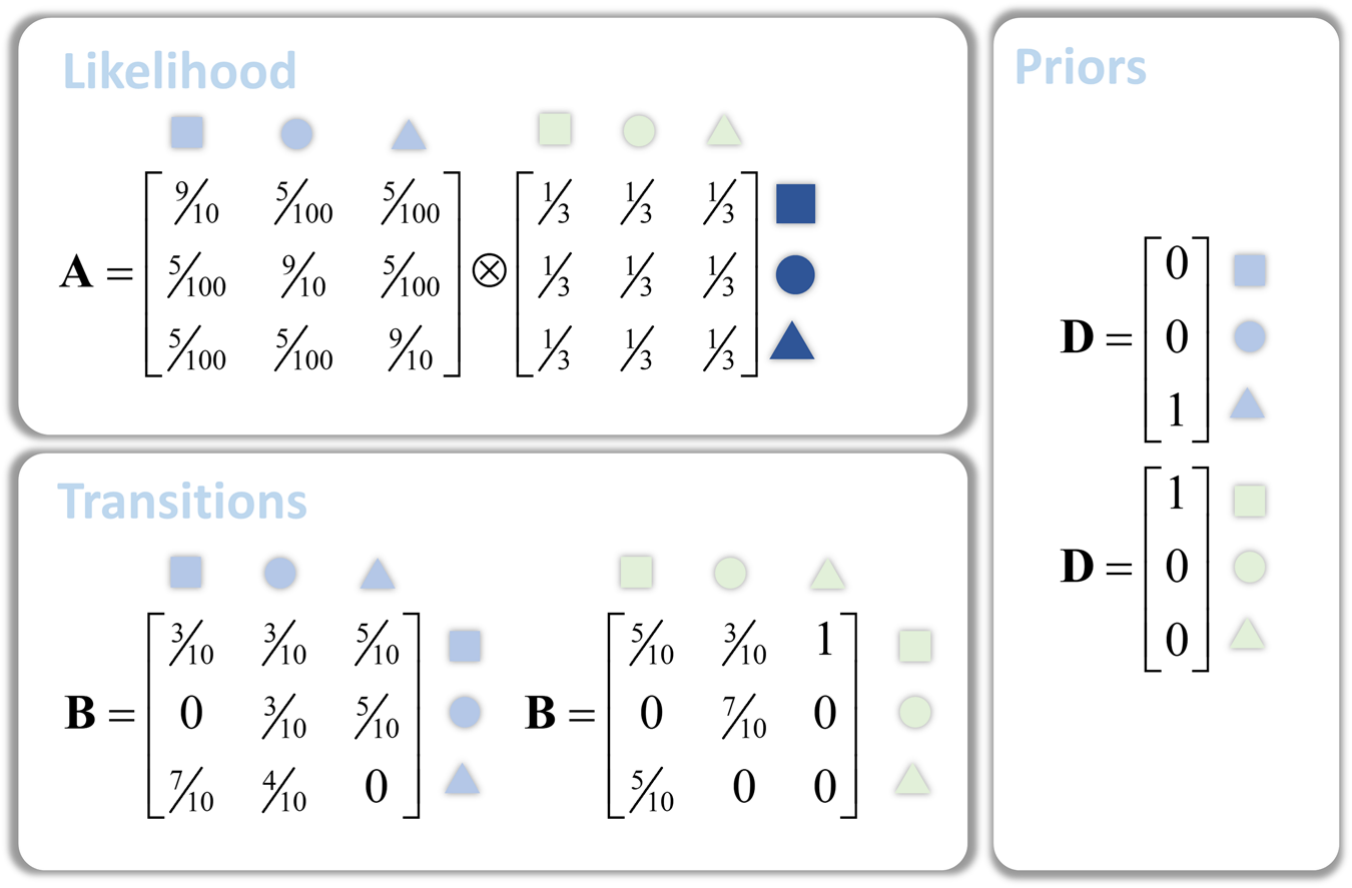
Probability distributions for simulated HMM. The probability distributions here make up the generative model we used to simulate neuronal message passing under both schemes. These probabilities have been chosen to make several points. Firstly, we separated the hidden state into two distinct state factors (light blue shapes and light green shapes). This allowed us to generate data (darker blue shapes) that depends upon only one of these (light blue). The likelihood mapping illustrates how data are (probabilistically) generated from these states ($P({o}_{\tau }=i|{s}_{\tau }=j)={{\bf{A}}}_{ij}$). We used deterministic priors for the initial states ($P({s}_{1}=i)={{\bf{D}}}_{i}$) and stochastic transitions ($P({s}_{\tau +1}=i|{s}_{\tau }=j)={{\bf{B}}}_{ij}$).

To compare the behaviour of each of these message passing schemes during online inference, we simulated their responses while presenting data sequentially. In other words, the scheme accumulates evidence for different hidden states by assimilating successive outcomes into posterior beliefs. We used an HMM employing the probability distributions specified in Fig. 8. This contains two hidden state factors (shapes of different colours) with data conditionally dependent upon only one. The purpose of this is to illustrate the behaviour of each scheme in the presence of informative and uninformative sensory input. Each of these hidden states starts with a defined shape (blue triangle, green square), but undergoes stochastic transitions. This means that the future should always be more uncertain than the past. In what follows, we use belief propagation as a gold standard for inferential performance, against which the other two schemes are compared. Our aim here is to illustrate the overconfidence of mean-field inference relative to the exact marginal inference of the Bethe approach, and to show how marginal approximations mitigate this, achieving a better approximation to belief propagation.

**Figure 9:**
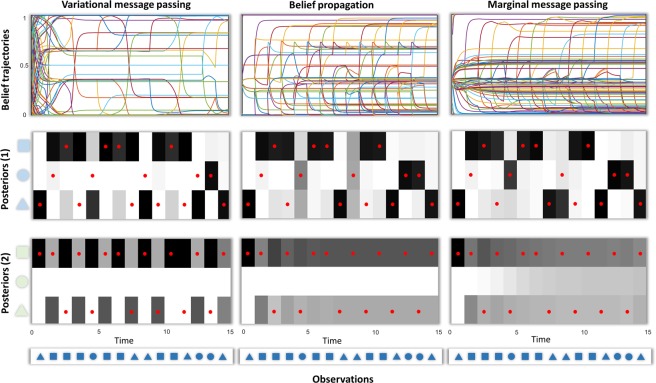
Simulated neuronal message passing for the three schemes: variational message passing, belief propagation and Marginal message passing. These plots show the consequences of generating data from the model described in Fig. 8 and solving the equations of Figs 5 and 6 for these data. The upper plots show the beliefs ($\tilde{{\bf{s}}}$) throughout the trial in terms of the sufficient statistics of the categorical distributions (i.e. probability of each alternative state at each time). These depict belief updating in terms of expectations about the two hidden factors, each with three levels. Crucially, these beliefs are about each hidden state at (15) different points in time. Each line is then the posterior probability that a given hidden state at a given time takes on a specific value. The colour-coding of these lines is consistent between the plots along the upper row. These plots are important in that they give a sense of the time-course of belief updating. While variational message passing shows rapid changes at the very start of the simulation (nearly every line reaches an extreme value within the first time-step) and few thereafter, the updates of belief propagation and marginal message passing happen over a much longer time scale. The beliefs after each successive outcome are shown in the second and third rows (with black = 1 and white = 0). Each line in the plots in the first row therefore represents a cell in the second or third row – drawn at the time point encoded by each line. Red dots indicate the ‘true’ states used to generate the data. Note that there are no red dots associated with green circles, as these never occur when the initial green state is a square (see the transition matrix in Fig. 8). The final row shows the sequence of observations presented to each message passing scheme. The second and third rows show the posterior beliefs about the two factors comprising the hidden state space as shown in Fig. 8.

Figure 9 shows the results of simulating inference via the three forms of neuronal message passing outlined above. This illustrates some cardinal features of the three schemes. The trajectories of beliefs following each outcome show that the majority of belief updating occurs very early in variational message passing, before the presentation of most of the data. While a few revisions to these beliefs occur at later stages, it does not take long to arrive at highly confident beliefs about future states – this over-confidence of posterior beliefs is a well-recognised feature of variational inference under the mean-field approximation^66^. In contrast, belief propagation and marginal message passing take a more restrained approach, with each new observation driving updating. This more tentative approach pays off, as they make fewer errors in estimating the true states that generated the data. This is consistent with the fact that belief propagation offers an exact estimate of marginal beliefs for these models, while the variational approach is only ever approximate.

The over-confidence of the variational approach manifests clearly in the posterior beliefs about the green shapes. Given the stochastic transitions, and the absence of any informative data about these states, posterior beliefs about the green shapes should become increasingly uncertain with distance from the (deterministic) initial state. The Bethe approach clearly shows this, but the variational scheme does not, with highly confident beliefs about even the penultimate state. Marginal message passing compensates for this overconfidence issue, providing a much better approximation to an exact inference scheme than under the mean-field approach. In fact, it slightly overcompensates in the absence of precise data, leading to posteriors that are less confident than the belief propagation marginals. The temporal dynamics of belief updating (the upper plots) further illustrate the overconfidence of variational message passing relative to the other two schemes. Within the first time-step, the sufficient statistics of beliefs about the states over time (each represented as a line) approach extreme (zero or one) values. This means that, with only one observation, the mean-field variational approach exhibits an excessive confidence about present and future states, that is maintained as new observations are made. In contrast, the belief propagation and marginal message passing schemes afford more modest belief updates – following the first observation – that become more confident as new data are acquired.

Notably, the three schemes share some of the same errors (four errors in steps 2, 4, 9 and 10). By errors, we mean that the inferred state (darkest shade) at a given time-step does not match the state that actually generated the data (red dot). These errors happen when very unlikely events occur, such as a dark blue square generated by a light blue triangle. Although incorrect, an inference that the light blue square caused the dark blue one is still Bayes optimal under the generative model we employed. In contrast, the additional four errors of variational message passing in steps 5, 8, 12 and 13 occur even though the data are highly consistent with the hidden states (e.g., a dark blue circle generated by a light blue circle). These errors reflect the excessive weight given to the empirical priors in variational message passing – it assumes the most probable *a priori* transition, ignoring the conflicting observation. It is important to note that increasing the dimensionality of the space state would further emphasise the failings of the mean-field approximation relative to the other two approximations.

To quantify the performance of the mean-field and marginal approaches, we can exploit the fact that the Bethe approach is exact for the marginal posteriors for this inference problem. A simple way to do this is to compute the KL-Divergence between the marginal posteriors obtained through belief propagation and the solutions of the other two schemes. The smaller this divergence, the better the approximation to exact marginal beliefs. For the simulations of Fig. 9, the divergences summed over marginal posteriors give the following:

$$\begin{array}{rcl}\sum _{\tau }{D}_{KL}[{Q}_{BP}({s}_{\tau })||{Q}_{VMP}({s}_{\tau })] & = & 86.0563\,nats\\ \sum _{\tau }{D}_{KL}[{Q}_{BP}({s}_{\tau })||{Q}_{MMP}({s}_{\tau })] & = & 3.7874\,nats\end{array}$$

This demonstrates quantitatively that, even for the relatively simple inference problem used here, there is a much greater divergence between the exact marginal posterior beliefs and those obtained using variational message passing, relative to marginal message passing.

Although we have presented this as a single simulation, the way in which the generative model is defined, and the sequential presentation of the data, actually induce several distinct inference problems that we have implicitly appealed to above in characterising these schemes. First, the factorisation of the hidden state-space into two different types of latent variable (the light blue and light green shapes of Figs 8 and 9) allows us to compare the extreme case in which data are uninformative about the latent variable (light green shapes) with the case in which there is only moderate uncertainty about the relationship between (light blue) states and the (dark blue) data. Figure 9 shows that, while marginal and Bethe approaches attenuate their confidence – when data is uninformative compared to informative – the mean-field approach furnishes confident inferences in both cases. Note that these differences rely upon there being some uncertainty in the transitions from one state to the next. If we were to use deterministic transition probabilities, the differences between these schemes would be largely abolished as all would make confident inferences.

The second comparison we have used relies upon sequential presentation of the outcomes. This means that each time-step represents a distinct inference problem, with more data available at later times than earlier. This is where the dynamics shown in the upper row of Fig. 9 are revealing. At each successive time point, the inference problem becomes more constrained, as an additional observation is made. This allows us to compare the confidence using a small amount of data (at the start of the trial) with the confidence after more data have been seen (near the end of the trial). After making the first observation, variational message passing shows a fairly consistent level of confidence until the end of the trial. This can be seen in the plot by noting that the distribution of lines in the vertical direction is relatively constant throughout the horizontal (temporal) axis. This contrasts with the other two schemes that show a greater proportion of lines reaching extreme values with each new observation.

## Discussion

This paper considers local computations that lend a biological plausibility to a range of inference schemes. We initially outlined two approaches to solving inference problems – variational message passing and belief propagation. Both can be expressed in a neurobiologically plausible form, but the former features a much simpler neuronal network structure. This simplicity comes at the cost of overconfidence, in comparison with the exact solutions obtained using belief propagation. The relative advantages of each motivated a third scheme; namely, ‘marginal message passing’ that uses a simple architecture but compensates for the problem of overconfidence. The temporal dynamics of inference reflect this, with variational message passing showing much earlier and exuberant changes in beliefs about those variables that are yet to give rise to observations, compared to the latter two schemes.

Crucially, we have expressed all three schemes in terms of differential equations describing evolution of beliefs over time (Figs 5 and 6). In addition to exposing the temporal dynamics of these approaches, and enabling a direct comparison, the resulting schemes also resemble the sorts of expressions that underwrite neural mass models^67^. In other words, the neuronal dynamics implied by all three schemes are determined by a mixture of the activity of other populations of neurons. This mixture of input determines the rate of change of a log probability, which may be thought of as a membrane potential. The membrane potential itself determines the influence of a neuronal population on other populations through a softmax function, and this can be seen as analogous to the translation of a membrane potential into an average firing rate. In short, all three schemes have a potential biological validity in that belief dynamics can be expressed in a form closely related to that of neuronal mass dynamics (please see^32^ for further details).

Note that the classical ‘forward-backward’ algorithm^68^ is a special case of the belief propagation applied to an HMM. This algorithm computes a set of forward probabilities and backwards probabilities before combining the results to give marginal posterior probability distributions. Marginal message passing can therefore be regarded as an approximation to the inference steps used in these schemes.

The ‘forward-backward’ algorithm (hence belief propagation) is also used in the inference step of the Baum-Welch algorithm^69^. However, the Baum-Welch algorithm has an additional step that distinguishes it from belief propagation and other variational approaches. This is a maximum likelihood update of the parameters of the generative model. The alternation between an inference step and a maximum likelihood update in the Baum-Welch algorithm underwrites its formal equivalence with the Expectation-Maximisation^70^ algorithm (as applied to an HMM), although the latter is rarely articulated in terms of message passing.

In contrast, the three variational schemes we have discussed here are (approximately) Bayesian, and so do not permit maximum likelihood updating. Instead, to draw inferences about parameters in these schemes, we need to specify prior distributions over the parameter values and use these to compute posterior beliefs^58,71^. From the perspective of Bayesian message passing, this just means extending the factor graph to include parameters, and passing additional messages. From the perspective of neurobiology, updates in beliefs about transition or likelihood probabilities would manifest as plastic changes in the efficacy of synapses connecting neuronal populations. This would allow for rewiring^72^ of the network to deal with a different (HMM) generative model if the way in which data were generated changed. Note that, once the generative model has been learned (as is assumed in the simulations here), the inference task depends only upon the neuronal activities, and does not require changes in connectivity in response to new data.

The particular form of message passing has implications for empirical studies, as disambiguating between alternative mechanisms that underwrite biological inference may be important in understanding psychopathology; e.g., hallucinations and delusions. All three message passing schemes make clear predictions about the time-course of electrophysiological responses at different time points following an outcome; i.e., within a trial. To adjudicate along different belief updating schemes, one could present participants with a sequence like that above, after exposing them to previous sequences so that they have learned the probabilistic structure of the task. If the brain employs variational message passing, we would anticipate greater evoked responses for the first few stimuli, given the greater rate of belief updating at these times. On immediate recall of the sequence, one might expect participants to make errors consistent with those found here – overestimating the precision of transitions.

To better accommodate behavioural responses, we could equip the schemes above with an active component^3^, and fit the resulting model to human behaviour^36^. It is more difficult to distinguish between belief propagation and a scheme (like marginal message passing) that seeks to emulate it in a simpler architecture. However, there are several forms of data that could be brought to bear on this question. First, one could appeal to a similar task as that above and fit the evoked responses with neural mass models^67^ that mimic the architectures of Figs 5 and 6. If additional neurons with fast time constants, representing the messages, improve the accuracy of the fit in excess of any increase in complexity, this would offer evidence in favour of belief propagation. Decreased accuracy, or preserved accuracy in the presence of an increased complexity, would instead favour a simpler architecture like marginal message passing. Further evidence could be garnered from tract tracing studies; e.g.^73^, or from single unit recordings – to ask whether they are better explained as representing messages rather than the sufficient statistics of marginal beliefs. Alternatively, one could compare the ability of simulated belief trajectories to explain electrophysiological responses. Current circuit-level research shows a high degree of consistency with the form of the neuronal networks presented here^74–76^, but these data are not sufficient to confidently disambiguate between the two architectures. Sensory input (via the thalamus) predominantly targets granular layers of cortex^77,78^, which excite more superficial cells and are disynaptically inhibited by them in turn^79^. This is consistent with Figs 5 and 6. The belief propagation architecture of Fig. 5 calls for another set of neurons (those representing the messages) that are inhibited by input from sensory streams and that excite the granular cells. Reversing the signs (excitation-inhibition), it is plausible that inhibitory interneurons in layer IV in receipt of sensory input^74^ could play this role; inducing an inhibition in the granular cells in response to sensory input (as opposed to exciting them in its absence). Apart from the empirical question, which of the neuronal schemes is supported by empirical data, there is also an important theoretical issue: we have focussed upon the neuronal manifestations of inference, but there are many other examples of biological inference that depend upon similar local interactions. It will be interesting to see whether the same principles of local message passing can be scaled up to collective behaviour^80,81^, with individuals exchanging information with their neighbours; or whether it can be scaled down to the computations performed by biochemical networks^82^.

Many neurological and psychiatric syndromes can be thought of in terms of false inference^83^. The form of healthy computation may be important for understanding the types of pathology that might affect it. Broadly speaking, computational pathologies can be described in terms of optimal inference using a suboptimal generative model^84–87^, or as broken inferential machinery^4,37^. An account of a pathology in terms of a suboptimal generative model transcends specific Bayesian message passing schemes and could be reproduced using variational message passing or belief propagation (or any other scheme). Theories of schizophrenia^86^, autism^88,89^, and visual neglect^87^ (among others) that appeal to pathological prior beliefs may be interpreted as deficits in any of the message passing schemes described here. In contrast, an account based on broken message passing commits to a specific neuronal implementation and is only meaningful if formulation of belief updating is correct.

An example of the latter is a recent theory that aims to account for inferential deficits that underwrite perceptual changes in schizophrenia. This posits ascending and descending inferential ‘loops’, and that disruption of these could lead to an ‘overcounting’ of a message^4^. To build some intuition for this idea, imagine we were to cut the starred connection in Fig. 5. This connection subtracts the ascending message from the forward message. A failure to subtract this message means the forward message will also contain the ascending message. The neurons representing the marginal then receive two copies of the ascending message; i.e., it is overcounted^37^. This could lead to an oversensitivity to sensory information^90^, and a failure to contextualise it using prior beliefs. That this is due to a failure of subtraction by an inhibitory interneuron is consistent with data suggesting disruptions in the balance of excitatory and inhibitory synaptic activity^91–93^ in patients with psychosis. Crucially, this account relies upon belief propagation implemented in a specific way – and does not generalise to marginal or variational message passing schemes.

## Conclusion

Variational message passing and belief propagation both represent means of performing Bayesian inference using local computations, consistent with the computations of biological neuronal networks. Both minimise free energy functionals, so are consistent with active inference – or the minimisation of free energy through action and perception. There are notable differences between the two schemes. While belief propagation represents exact Bayesian inference (for many generative models), variational approaches yield approximate inference. The latter tends towards excessive confidence in the face of uncertainty, deviating from exact Bayesian optimality. However, the kinds of neuronal network that support belief propagation appear to require a greater number of neurons and axons to achieve biological plausibility. This suggests a trade-off between inferential accuracy (belief propagation) and a complexity cost (variational message passing). Drawing from the relative benefits and drawbacks, we have introduced a third possibility. The brain may approximate belief propagation, using circuitry consistent with variational message passing. We hope to disambiguate between these possibilities in future work. An interesting conceptual issue that arises from these considerations is how best to understand the false inferences that underwrite neurological and psychiatric disease. Appealing to broken generative models enables one to be agnostic about the exact form of message passing, while hypothetical lesions to the inferential machinery depend upon the validity of that machinery.

## Supplementary information

Appendix

## Data Availability

The script used for the simulations is available at https://github.com/tejparr/nmpassing.
