# Supplementary material for: Neuronal message passing using Mean-field, Bethe, and Marginal approximations
Source: Sci Rep. 2019 Feb 13;9:1889. doi: 10.1038/s41598-018-38246-3 (PMC6374414; doi:10.1038/s41598-018-38246-3)
Supplement: Supplementary file 1 — Appendix [file 41598_2018_38246_MOESM1_ESM.docx]

Neuronal message passing using

Mean-field, Bethe, and Marginal approximations

Thomas Parr^1^, Dimitrije Markovic^2^, Stefan J. Kiebel^2^, Karl J Friston^1^

^1^ Wellcome Centre for Human Neuroimaging, Institute of Neurology, University College London, WC1N 3BG, UK.

^2^ Chair of Neuroimaging, Psychology Department, Technische Universität Dresden, Dresden, Germany

[thomas.parr.12@ucl.ac.uk](mailto:thomas.parr.12@ucl.ac.uk), [dimitrije.markovic@tu-dresden.de](mailto:dimitrije.markovic@tu-dresden.de), stefan.kiebel@tu-dresden.de, [k.friston@ucl.ac.uk](mailto:k.friston@ucla.ac.uk)

**Correspondence**: Thomas Parr

The Wellcome Centre for Human Neuroimaging

Institute of Neurology

12 Queen Square, London, UK WC1N 3BG

[thomas.parr.12@ucl.ac.uk](mailto:thomas.parr.12@ucl.ac.uk)

**Appendix – Free energy minimisation**

*Variational free energy*

Variational message passing is obtained simply by setting the variation of the free energy with respect to a marginal posterior equal to zero ^66^.

$$\begin{matrix} F=-\sum_{\tau} (E_{Q(s_{\tau})Q(s_{\tau-1})}[\ln P(s_{\tau}|s_{\tau-1})]+E_{Q(s_{\tau})}[\ln P(o_{\tau}|s_{\tau})])-\sum_{\tau} H[Q(s_{\tau})] \\ \delta_{Q(s_{\tau})}F=0\Leftrightarrow Q(s_{\tau})=\sigma(\ln P(o_{\tau}|s_{\tau})+E_{Q(s_{\tau-1})}[\ln P(s_{\tau}|s_{\tau-1})]+E_{Q(s_{\tau+1})}[\ln P(s_{\tau+1}|s_{\tau})]) \end{matrix}$$

*Bethe free energy*

Belief propagation is slightly more complicated to obtain ^26^. This requires the additional constraint that $h_{\tau-1,\tau}=\sum_{s_{\tau-1}} Q(s_{\tau},s_{\tau-1})-Q(s_{\tau})=0$ (i.e. that summing over the pairwise factors gives the marginal). Introducing Lagrange multipliers, we can find the free energy minimum subject to this constraint.

$\begin{matrix} F=-\sum_{\tau} (E_{Q(s_{\tau},s_{\tau-1})}[\ln P(s_{\tau}|s_{\tau-1})]+E_{Q(s_{\tau})}[\ln P(o_{\tau}|s_{\tau})]) \\ -\sum_{\tau} (H[Q(s_{\tau})]-D_{KL}[Q(s_{\tau},s_{\tau-1})||Q(s_{\tau})Q(s_{\tau-1})]) \end{matrix}$

$\begin{matrix} \delta_{Q(s_{\tau})}(F+\alpha(s_{\tau})h_{\tau-1,\tau}+\beta(s_{\tau})h_{\tau+1,\tau})=0\Leftrightarrow Q(s_{\tau})=P(o_{\tau}|s_{\tau})e^{-\alpha(s_{\tau})}e^{-\beta(s_{\tau})} \\ \delta_{Q(s_{\tau},s_{\tau-1})}(F+\alpha(s_{\tau})h_{\tau-1,\tau}+\beta(s_{\tau})h_{\tau-1,\tau-1})=0\Leftrightarrow Q(s_{\tau},s_{\tau-1})=Q(s_{\tau})Q(s_{\tau-1})P(s_{\tau}|s_{\tau-1})e^{\alpha(s_{\tau})}e^{\beta(s_{\tau-1})} \end{matrix}$

Solving for the Lagrange multipliers,

$$\begin{matrix} e^{-\alpha(s_{\tau})}=\sum_{s_{\tau-1}} P(o_{\tau-1}|s_{\tau-1})P(s_{\tau}|s_{\tau-1})e^{-\alpha(s_{\tau-1})} \\ e^{-\beta(s_{\tau})}=\sum_{s_{\tau+1}} P(o_{\tau+1}|s_{\tau+1})P(s_{\tau+1}|s_{\tau})e^{-\beta(s_{\tau+1})} \end{matrix}$$

Equating $e^{-\alpha(s_{\tau})}=\mathop{\mu_{B}}\limits^{\to} (s_{\tau})$ and $e^{-\beta(s_{\tau})}=\mathop{\mu_{B}}\limits^{\leftarrow} (s_{\tau})$, we see that this is identical to belief propagation.

*Marginal free energy*

To derive the marginal message passing scheme, we set the variational derivative of the marginal free energy at each time step to zero.

Note that the halving of the contribution from the future and the past implies that, for any non-deterministic transitions, we deliberately over-estimate the volatility (imprecision of transitions) of the model. This effectively compensates for the overconfidence problem in variational message passing, and probably over-compensates.
